# Colorectal Cancer Screening Modalities Among Saudi Population: Significant Predictors

**DOI:** 10.2174/0115748871335743250417103659

**Published:** 2025-05-05

**Authors:** Mohammad Bukhetan Alharbi

**Affiliations:** 1 General Surgery, Department of Surgery, Medical College, Imam Mohammad Ibn Saud Islamic University (IMSIU), Riyadh, Saudi Arabia

**Keywords:** Colorectal, screening, fecal immunochemical test, cancer, public, modality

## Abstract

**Introduction:**

Understanding the public perception of colorectal cancer screening is crucial since early diagnosis lowers mortality rates associated with this type of cancer.

**Methods:**

The purpose of this study was to determine the most popular technique for colorectal cancer screening in Saudi Arabia. From September 2021 to February 2022, a cross-sectional study on adult Saudi population was undertaken. Data were collected through a self-created survey distributed *via* an online platform.

**Results:**

Out of 10,781 participants analyzed, 5,612 were found suitable for evaluating preferences between colonoscopy and fecal immunochemical test (FIT) as screening modalities. It was found that gender, employment, and age were important predictors for the preferred screening modality.

**Discussion:**

The patient’s perspective on screening modalities is crucial to consider in national colorectal cancer screening programs. The future outlook for home-based fecal immunochemical Tests (FIT) may provide superior research regarding cost-effectiveness and adherence among patients compared to colonoscopy or medical facilities in FIT investigations.

**Conclusion:**

More attention should be paid to the population’s preferred method of colorectal cancer screening, which could increase the success of the screening effort. Cultural and socioeconomic status might affect the generalizability of the study.

## 1 INTRODUCTION

Colorectal cancer (CRC) remains one of the leading causes of cancer-related morbidity and mortality worldwide. Early detection through screening is crucial in improving survival rates, and various screening modalities have been developed to facilitate this. Understanding the significant predictors that influence the choice and effectiveness of these screening methods is essential for optimizing colorectal cancer prevention strategies.

Colorectal cancer ranks as the second most common cancer-related cause of death globally. While industrialized nations have a high mortality rate as a result of the disease, developing nations have also experienced a surge in the rate of colorectal cancer-related deaths over the past decade [1]. Screening with a high-sensitivity guaiac-based fecal occult blood test revealed a 4.8% prevalence of colorectal cancer in Saudi Arabia [2]. Alarmingly, over 60% of the population is unaware of the symptoms of colorectal cancer [3]. It is essential to take into account the rise in early-onset colorectal cancer cases in Saudi Arabia [4].

Patient impression of the type of screening modalities is highly essential, even though such perceptions may not directly correlate with utility, as is shown by the diverse screening programs implemented across continents [5]. When it comes to colorectal cancer screening, cost-effectiveness must be assessed from the payer's perspective. Studies indicate that the fecal immunochemical test (FIT) performs better than colonoscopy as the initial screening test [6]. Other modalities, such as fecal DNA tests, are also employed if efforts to provide the standard screening are hampered [7].

## 2 METHODOLOGY

In this descriptive cross-sectional study, the Saudi Arabian population was examined, with data collection conducted between November 2021 and March 2022. A self-administered survey was administered randomly to the general public *via* social media platforms. The midyear population of Saudi Arabia in 2020 was used to calculate the sample size through the level of precision formula with a margin error of 5% and a confidence level of 95%, thereby yielding a sample size of 385. There were a total of 10,781 participants in this study. Before the survey began, all respondents were provided with a website outlining the goals of the research and were requested to provide their consent. To prevent duplicate responses, each participant’s questionnaire was linked to network technology. This study protocol was authorized by the Medical College Institutional Review Board of Al-Imam Mohammad Ibn Saud Islamic University in Riyadh, Saudi Arabia, under project number 99-2021.

### 2.1 Criteria for Inclusion and Exclusion

This survey targeted the general population in Saudi Arabia who could read and write in Arabic (the main language of Saudi Arabia) and were 18 years or older. The study excluded those living outside Saudi Arabia, those less than 18 years old, and those with a diagnosis of CRC. Participants’ responses were collected from their social media accounts on Twitter. Duplicate responses were eliminated.

### 2.2 Data Collection Tool

The study utilized a self-designed survey of twenty-two items, thirteen of which asked about socio-demographic factors and the remaining nine concerning the CRC screening preferences of the general population of Saudi Arabia. The questionnaire was validated by two subject experts and the pilot study was conducted for a sample size of twenty. The reliability coefficient Cronbach’s alpha for the questionnaire was estimated to be 0.889.

## 3 OVERVIEW OF COLORECTAL CANCER SCREENING MODALITIES

There are several screening modalities for colorectal cancer, each with its strengths and weaknesses, which are as follows:

### 3.1 Colonoscopy

This invasive procedure allows for direct visualization of the colon and rectum, enabling both diagnosis and removal of polyps. It is considered the gold standard for colorectal cancer screening. However, its high cost, the need for bowel preparation, and potential complications can serve as barriers.

### 3.2 Fecal Immunochemical Test (FIT)

It is a non-invasive stool test that detects hidden blood in the feces, indicating potential cancer. FIT is less invasive and more cost-effective than colonoscopy, but it may miss polyps that are not bleeding.

### 3.3 Computed Tomography Colonography (CTC)

Also known as virtual colonoscopy, this imaging technique provides a non-invasive alternative to traditional colonoscopy. It can visualize the entire colon, but requires bowel preparation and cannot remove polyps.

### 3.4 Stool DNA Tests

These tests analyze stool samples for genetic markers associated with colorectal cancer. They offer convenience and ease of use, but may not be as widely available or may require follow-up colonoscopy if positive.

### 3.5 Sigmoidoscopy

This procedure examines only the lower part of the colon. While effective for detecting left-sided cancers, it may miss lesions in the proximal colon.

Among the above methods, the first two (colonoscopy and fecal immunochemical test) are the most common ones and were considered in the current study.

## 4 SIGNIFICANT PREDICTORS OF SCREENING MODALITIES

Several predictors influence the choice of screening modality and the likelihood of participation in colorectal cancer screening. They are described as follows:

### 4.1 Age and Gender

Age is one of the most significant predictors, as screening recommendations generally begin at age 45 for average-risk individuals [8]. Studies show that participation rates tend to be higher among older adults. Gender also plays a role, with some studies indicating that women may be less likely to undergo screening than men, potentially due to differing health-seeking behaviors.

### 4.2 Socioeconomic Status

Socioeconomic factors significantly impact access to healthcare and screening services. Individuals with higher income and education levels are more likely to engage in regular screening. Barriers, such as lack of insurance, transportation issues, and the cost of procedures, can deter lower-income populations from accessing CRC screening [9].

### 4.3 Health Literacy

Health literacy affects individuals' understanding of the importance of screening, the various modalities available, and the procedures involved. Those with higher health literacy are more likely to participate in screening and select appropriate modalities based on their risk factors [10].

### 4.4 Family History and Genetic Predisposition

A family history of colorectal cancer or the presence of hereditary syndromes, such as Lynch syndrome or familial adenomatous polyposis (FAP), significantly increases an individual's risk of developing CRC. Those with a family history are often more proactive in seeking screening, and they may prefer more invasive modalities, like colonoscopy, for thorough examination [10].

### 4.5 Comorbid Conditions

Patients with comorbid conditions may have different preferences for screening modalities based on their overall health status. For instance, individuals with significant health issues may be reluctant to undergo invasive procedures, like colonoscopy, due to the associated risks. Conversely, those in good health may opt for more comprehensive screening methods [11].

The current study considered age, gender, and employment status as the predictors of modalities of CRC.

## 5 STATISTICAL ANALYSIS

The continuous variables have been presented as mean (SD)/median (IQR) and the categorical variables have been presented as frequency and percentage. The chi-square test was used to find the association between categorical variables. Univariate logistic regression was performed to find the significant predictors of modalities of CRC screening. The variables age, gender, region, nationality, education status, monthly income, and family history were the characteristics used to predict modalities of CRC screening. Among these variables, age, gender, and employment status were statistically significant and used for the multivariate logistic regression analysis. All the analyses were performed using IBM SPSS version 22 and the level of statistical significance was decided based on a *p*-value less than 0.05.

## 6 RESULTS

In the subgroup analysis of the main study [12], 5,570 subjects were involved and the sociodemographic characteristics of the subjects are displayed in Table **1** and Figs. (**1** and **2**). The majority of the subjects (58%) were in the 18-25 years of age category and less than 1% were in the 70 years of age and above category. Compared to males, female subjects were more in number.

### 6.1 Association between CRC Screening Modalities and Demographic Variables

The chi-square test was used to find the association between CRC screening modalities and demographic variables. The age groups were recoded into two groups, below 40 and above 40, and there was a statistically significant association between age and screening modality (*p* <0.001). The younger age group (below 40) was more likely to choose an immunochemical test (62.2%) compared to colonoscopy (37.8%). In the age group above 40, 53.6% preferred an immunochemical test, and 46.4% preferred a colonoscopy.

In the case of gender, there was also a statistically significant association between gender and screening modality (*p* =0.014). Among the male subjects, 58.7% preferred immunochemical test and 41.3% chose colonoscopy. Among female subjects, 61.8% selected the immunochemical test, while 38.2% preferred colonoscopy.

Employment status and screening modality also showed a statistically significant association (*p* =0.008). Among employed individuals, 58.7% preferred immunochemical test and 41.3% chose colonoscopy. Among unemployed subjects, 62% selected the immunochemical test, while only 38% chose colonoscopy (Tables **2**-**5**).

### 6.2 Logistic Regression Analysis

Multivariate logistic regression analysis was performed to find out the significant predictors of screening modalities. The fecal immunochemical test was taken as the reference variable for the logistic regression analysis in comparison with colonoscopy. The variables age (2 categories, below 40 and above 40), gender, and employment (2 categories, employed and unemployed) were used as predictors.

## 7 DISCUSSION

According to studies, three-quarters of the population prefers fecal immunochemical testing over colonoscopy for colorectal cancer screening [13], which makes our results consistent with past findings [12]. The optimal screening method may rely on a variety of variables, such as the willingness to invest in quality-adjusted life years, the availability of endoscopy services, and the economic impact [14]. However, 50-55% of individuals cited fear of uncomfortable colonoscopy procedures and a lack of understanding about the accessibility of the fecal immunochemical test as barriers to screening. Strong gender disparities were noted, with females having higher overall and colonoscopy-specific obstacles to colorectal cancer screening than men [15].

Inadequate public understanding of colorectal cancer and its preventative measures was found to be primarily related to a lack of education [16]. It is recommended to design countrywide evidence-based strategies and programs that take into account the proximity of primary care clinics for the elderly and enhance the ease of mobility [17].

The results also suggested that the onset of colorectal cancer in the Saudi population occurs earlier than in the West, specifically between the ages of 44 and 50. On average, Saudi women and men are diagnosed with colorectal cancer at ages 56 and 60, respectively. This is notably younger than in the United States, where the comparable figures are 71 and 67 years. This provides indications that colorectal cancer occurs at an earlier age in the Saudi population. Hence, adenoma prevalence should correspond with these data, particularly in the age categories of 41-45 and 46-50 years [18].

According to reports, 70% of the population expressed willingness to undergo colorectal cancer screening [19]. However, perceived burdens significantly limit access to colon cancer screening. Budgetary constraints can affect both cancer therapy and cancer screening, highlighting the need for healthcare providers to be mindful of these barriers [20].

Interestingly, a positive fecal immunochemical test is connected with a heightened incidence of rheumatoid arthritis in the community, verifying that gut mucosal abnormalities are linked to the onset of systemic lupus erythematosus, rheumatoid arthritis, and psoriatic arthritis. As a result, individuals in this population require close monitoring and prompt referral to specialists when clinical concerns arise [21].

Infrequently, adenomas could be the source of a positive FIT result. However, the mechanisms behind their detection by FIT remain unclear. Furthermore, a normal endoscopy following a positive FIT result does not rule out the possibility of detecting severe tumor formation in the subsequent screening [22]. The selected FIT method minimized the disparity in diagnostic efficiency between males and females, particularly in the diagnosis of CRC. Excellent participation rates were observed, leading to moderate positive levels and detection rates. The results have indicated that gender-specific methods can be implemented in CRC screening programs [23].

In a national FIT diagnostic program, a follow-up FIT should be planned following a normal colonoscopy to detect overlooked tumors and lower the risk of occurrence of CRC [24]. Incorporating patient opinions about their screening modalities is quite essential for the success of national programs for CRC screening. Trends suggest that home-based FIT options may prove even more effective in terms of cost and patient compliance as compared to colonoscopy or hospital-based FIT.

## CONCLUSION

Understanding the population’s preferences regarding the type of CRC screening is crucial for achieving more successful results and improving overall public health. Our results have indicated that the fecal immunochemical test shows promise as a more effective and widely accepted option for CRC screening programs compared to colonoscopy, due to its ease of use, cost-effectiveness, and potential for higher compliance rates.

## STUDY LIMITATIONS

Cultural and socioeconomic factors of the community may have a different view of the preferred type of screening.

## Figures and Tables

**Fig. (1):**
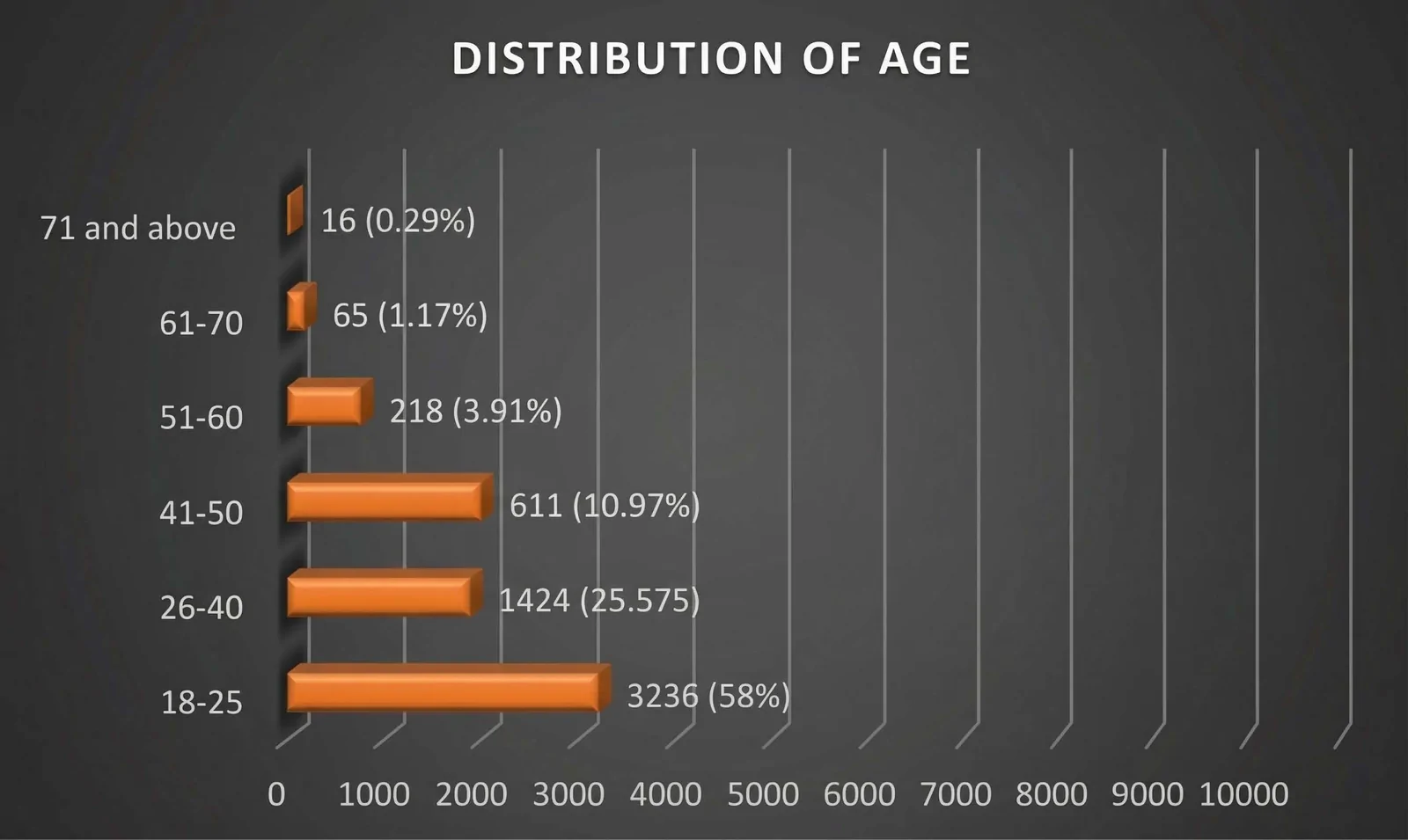
Distribution of age.

**Fig. (2):**
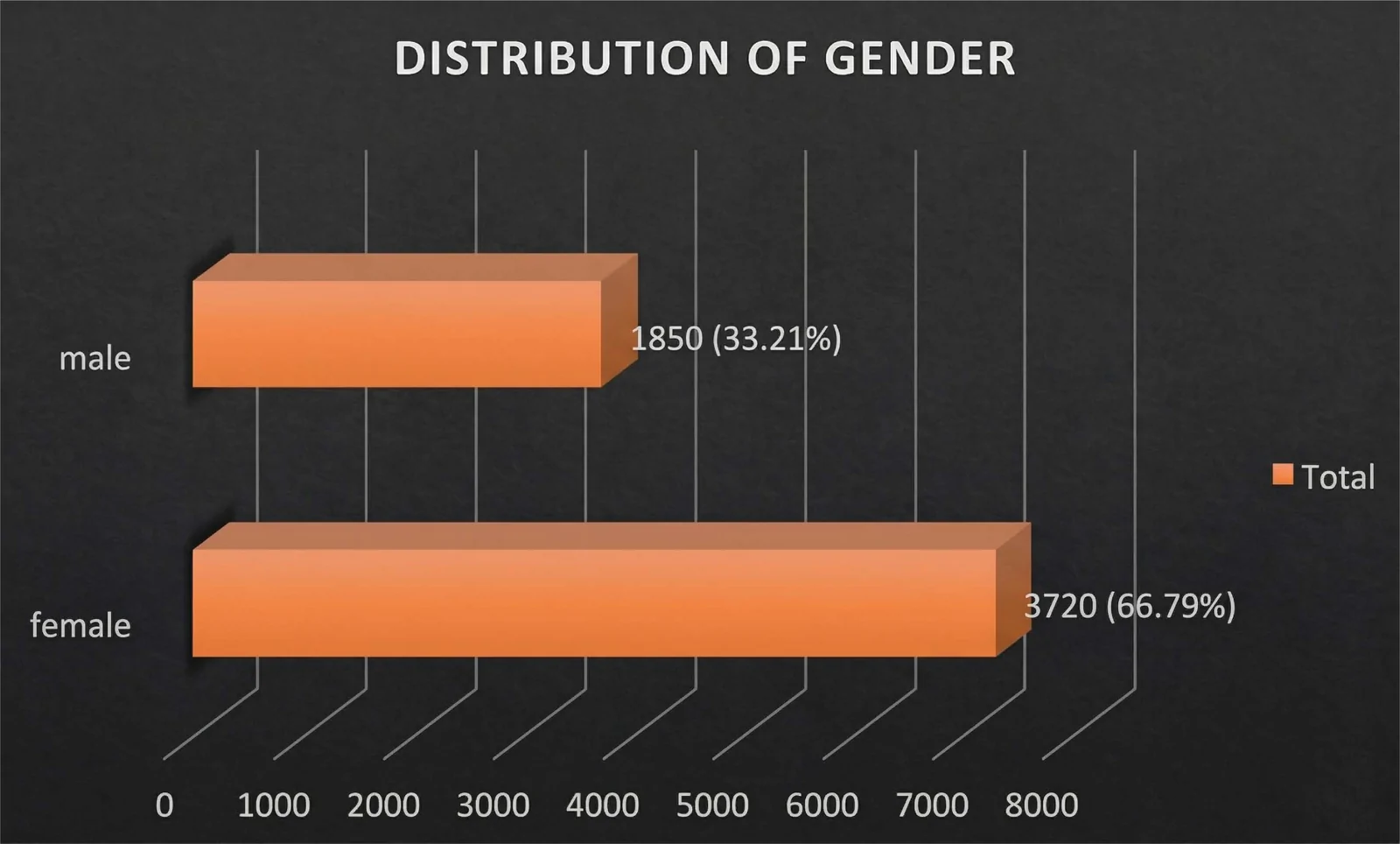
Distribution of gender.

**Table 1:** Socio-demographic characteristics of the subjects (*n*=5570).

**Variables**	**N (%)**
**Age group**	**-**
18-25	3236 (58)
26-40	1424 (25.57)
41-50	611 (10.97)
51-60	218 (3.91)
61-70	65 (1.17)
71 and above	16 (0.29)
**Gender**	**-**
Male	1850 (33.21)
Female	3720 (66.79)
**Region**	**-**
Eastern	934 (16.77)
Middle	879 (15.78)
Northern	1175 (21.1)
Southern	1407 (25.26)
Western	1175 (21.1)
**Nationality**	**-**
Saudi	5335 (95.78)
Non-Saudi	235 (4.22)
**Education level**	**-**
Bachelor’s degree	3352 (60.18)
Health practitioner	216 (3.88)
High school	1653 (29.68)
Master's degree, PhD, and above	227 (4.08)
Primary	122 (2.19)
**Employment status**	**-**
Employed full-time	1512 (27.14)
Employed part-time	267 (4.79)
Self-employed	262 (4.71)
Unemployed	3529 (63.36)
**Monthly income**	**-**
12000-25000	711 (12.77)
6000-12000	1096 (19.68)
Less than 6000	3614 (64.88)
More than 25000	149 (2.68)
**Family history of cancer**	**-**
Yes	-
No	-

**Table 2:** Association between age and screening modalities.

Age * Screening_modality Crosstabulation						***P*-Value**
**-**			**Screening_modality**		**Total**	
			**Immunochemical Test**	**Colonoscopy**		
**Age**	Below 40	n	2923	1774	4697	<0.0001
		%	62.2%	37.8%	100.0%	
	Above 40	n	490	425	915	
		%	53.6%	46.4%	100.0%	
Total		-	3413	2199	5612	
		-	60.8%	39.2%	100.0%	

**Table 3:** Association between gender and screening modalities.

Gender * Screening_modality Crosstabulation						***P*-Value**
**-**			**Screening_modality**		**Total**	
			**Immunochemical Test**	**Colonoscopy**		**-**
**Gender**	Male	n	1095	769	1864	0.014
		%	58.7%	41.3%	100.0%	
	Female	n	2318	1430	3748	
		%	61.8%	38.2%	100.0%	
Total			3413	2199	5612	
			60.8%	39.2%	100.0%	

**Table 4:** Association between employment and screening modalities.

**-**						***P*-Value**
**-**			**Screening_modality**		**Total**	
			**Immunochemical Test**	**Colonoscopy**		
**Employment**	Employed	1211		851	2062	0.008
		58.7%		41.3%	100.0%	
	Unemployed	2202		1348	3550	
		62.0%		38.0%	100.0%	
Total		3413		2199	5612	
		60.8%		39.2%	100.0%	

**Table 5:** Logistic regression model.

**Variables**	**B**	**SE**	**OR**	**95% CI**	***P*-Value**
Gender	-.082	.061	.921	(.816, 1.039)	.181
Employment	-.035	.062	.965	(.854, 1.091)	.572
Age	.329	.076	1.390	(1.197, 1.614)	<0.001

## Data Availability

The data and supportive information are available within the article.

## LIST OF ABBREVIATIONS

CRC: Colorectal Cancer
CTC: Computed Tomography Colonography
FAP: Familial Adenomatous Polyposis
FIT: Fecal Immunochemical Test

## STANDARDS OF REPORTING

STROBE guidelines were followed.
